# Ultrasound Diagnosis and Follow-Up of Neonate Renal Candidiasis

**DOI:** 10.5334/jbr-btr.1059

**Published:** 2016-12-20

**Authors:** Yves Boulanger, Marie-Sophie Ghuysen, Alain Nchimi, Michel Lewin, Jamil Khamis

**Affiliations:** 1 CHC Liège, Belgium, BE; 2 CH Luxembourg, LU

**Keywords:** Neonate, Kidney, Candidiasis, Ultrasonography

## Abstract

Urinary tract infection by Candida Albicans is a severe condition that can occur in infants during the course of a preterm or intensive care hospitalization. Candidiasis can affect the kidney and dramatically impair renal function through involvement of the renal cortex, typically associated with potentially obstructive pelvi-caliceal fungus balls. This case report describes the case of a 4.5-month girl who developed renal candidiasis one week after her admission for upper respiratory tract infection. To manage the risk of urinary tract obstruction by fungus balls, several options were discussed and a conservative approach preferred to surgery that has a potential long-term impact on renal growth, proved effective.

## Case Report

A 4.5-month-old girl was admitted to the emergency room for loss of consciousness. Her past nine days were marked by cough and apathy. She was born at 41 weeks with a weight of 3.5 kg and her past history was unremarkable. Physical examination was strictly normal and the patient was looking good, with only a mild cough. The diagnosis of respiratory syncytial virus infection was made by Polymerase Chain Reaction. The rest of the blood analysis was strictly normal, with no inflammatory syndrome. In response to a rapid decrease of arterial oxygen saturation in the emergency room, the baby was transferred to intensive care, intubated and her bladder catheterized to monitor liquid excretion. Broad-spectrum antibiotherapy was started to prevent bacterial surinfection. After three days of intubation, the patient presented oliguria and acute renal failure with transient peaks of elevated blood pressure (120/65 mmHg, treated with diuretics) and fever (38.6°C). Blood culture and lumbar puncture detected neither bacteria nor fungal agent. Meanwhile, pyuria was detected and Candida Albicans was present in urinary cultures above the threshold of 100,000 colony-forming units per milliliter. A treatment by intravenous Fluconazole was started. The subsequent immune check-up detected no anomaly, and a renal ultrasound was requested.

**Figure 1:**
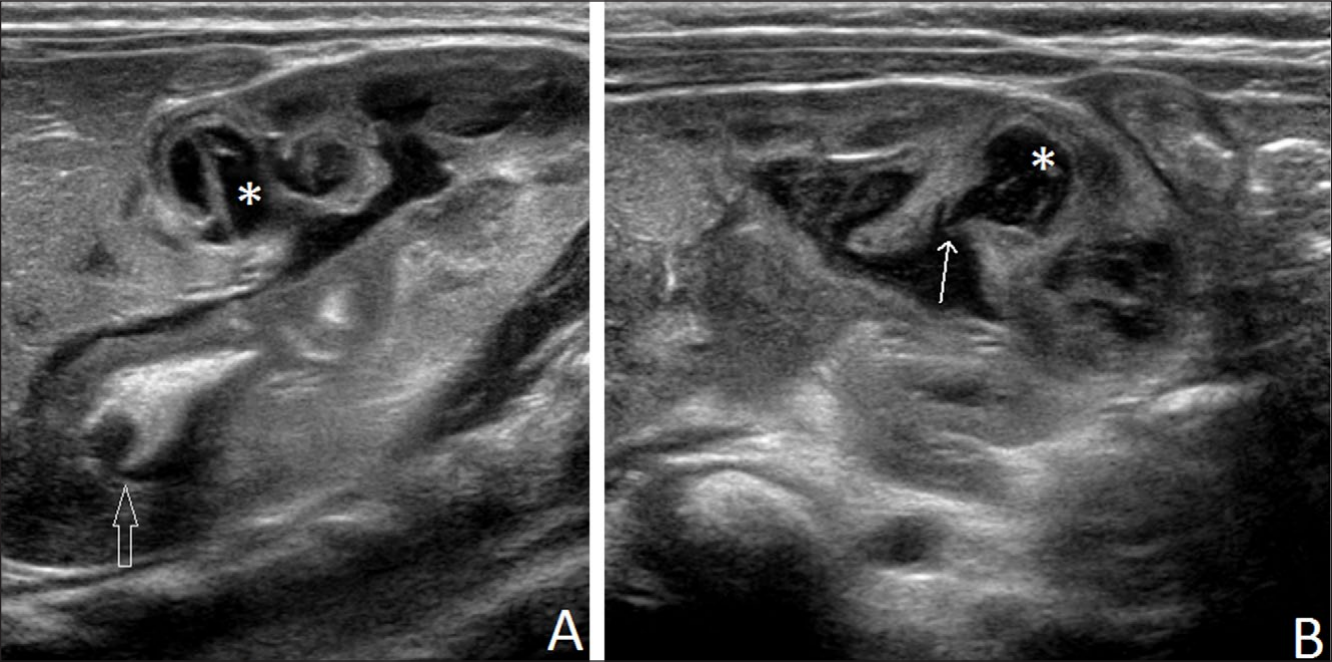
**(A)** Longitudinal ultrasound scan of the right kidney shows abscesses (asterisk), where at some locations echoic trident-shaped fungus balls (open arrow) are seen. **(B)** There are communications (arrow) between abscesses and dilated pelvi-caliceal cavity (asterisk).

**Figure 2:**
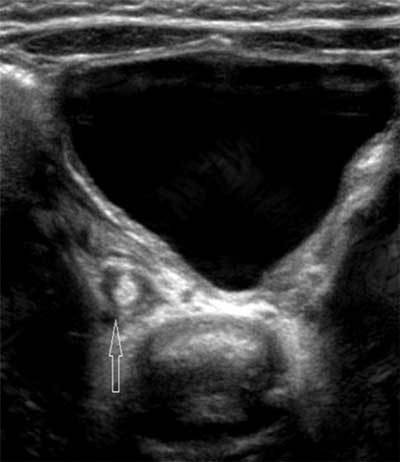
Axial ultrasound scan of the right pelvic ureter containing a fungus ball (open arrow).

It showed a right nephromegaly with hyperechoic parenchyma. There were five parenchymal anechoic rounded formations with septations and internal echoes. Some of them were connected to the pelvi-calyceal system through a thin meatus. Pelvi-caliceal wall was thickened and the cavities contained rounded hyperechoic nodules suggesting fungus balls, with mild dilatation of some calices, and hyperechoic formations with fork shape suggesting moulded image of papillae (Figure 1). Because of the right urinary tract obstruction (Figure 2) an the potential risk of subsequent renal failure, the question of open-kidney surgery was discussed. Considering the risk for the kidney long-term growth after surgery, we decided for a conservative treatment, which consisted of 3 months of Fluconazole.

**Figure 3:**
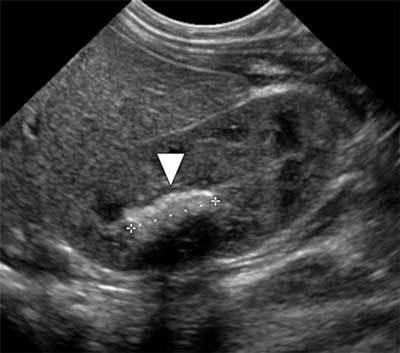
Follow-up ultrasonography shows calcification in the right kidney with posterior shadowing (arrowhead).

Follow-up ultrasound examinations showed a favorable evolution, with shrinking of the abscesses and increasing acoustic shadowing of the echoic material in the calices suggesting calcification (Figure 3). Iterative urinary culture showed decrease in pyuria. The baby suffered then two nephritic colic episodes due to elimination of calcified fragments, requiring pain management, respectively at the age of 5.5 and 6 months, confirmed by ultrasonography. There has been no reinfection yet, 6 months after the release from the hospital.

## Discussion

Fungal skin infection is common in newborns. However, it rarely progresses to a systemic form, unless certain risk factors are present [1]. Candida albicans is the most frequently implicated agent in systemic candidiasis [2]. Candida albicans has an affinity for the heart, central nervous system, kidney and eye. Renal candidiasis is the most common systemic form [3].

Renal candidiasis has nonspecific clinical presentation and may be revealed by hypertension or acute renal failure in obstructive syndrome [3], as in our case. Blood cultures are often negative and the diagnosis of renal candidiasis is usually made on the isolation of Candida in urine culture. In case of negative urinary culture, looking for the specific antigen can help [3]. Ultrasound is the modality of choice to monitor the infection [3].

Renal ultrasound may show two overlapping patterns [3,4]. Parenchymal involvement is associated with increased volume and hyperechoic renal cortex, similar to pyelonephritis. Involvement of the cortex is focal or global [1]. Septate cavities may be present in the cortex corresponding to abscesses caused by ischemia resulting from obstruction by fungal balls. These have a low echoic or anechoic content, corresponding to collected pus or cavities self-draining into the pelvi-caliceal cavities, as in the case reported. On the other hand, germ excretion in the excretory tract results in fungal balls, which appear hyperechoic and rounded, without acoustic shadowing. They result from the aggregate of mycelial fragments [5] and can obstruct the urinary tract. Fungal balls must also be found in the ureters, bladder and urethra [1]. Once treated they often calcify and gradually disappear, as in this case [1,5]. They can be confused with necrotic papillae, blood clots, nephrocalcinosis or tumor, and clinical information is crucial. Conservative treatment is based on systemic antifungal fluconazole or amphotericin B [4]. There is no evidence-based recommendation. A pragmatic approach is to continue until urine sterilization. The treatment can be extended from three to six weeks in case of parenchymal disease [3]. It should be emphasized that ultrasound appearance is not correlated with the healing, and fragmented or calcified sterile fungal balls can still be seen at the end of treatment [1,3]. The kidney ultrasonography objective is to exclude obstructive syndrome, possibly requiring drainage or surgery [3,4]. In this case, mild dilatation of some calices suggested potential deleterious long-term consequences on the function of the kidney. But the follow-up showed stable then decreasing dilatation of the calyces.

Computed tomography is superior to ultrasonography in the study of pyelonephritis and abscess. It allows better visualization of perinephric fluid, gas and fungus balls. However, it has a minor role in pediatric imaging, owing to radiation dose issues. MRI with gadolinium enhancement would be a good alternative, as it surpasses scintigraphy in demonstrating febrile urinary tract infection in children [6].
